# Chainchecker: An application to visualise and explore transmission chains for Ebola virus disease

**DOI:** 10.1371/journal.pone.0247002

**Published:** 2021-02-19

**Authors:** Katy Gaythorpe, Aaron Morris, Natsuko Imai, Miles Stewart, Jeffrey Freeman, Mary Choi

**Affiliations:** 1 Imperial College London, London, United Kingdom; 2 University of Cambridge, Cambridge, United Kingdom; 3 Applied Physic Laboratory, Johns Hopkins University, Baltimore, Maryland, United States of America; 4 Centers for Disease Control and Prevention, Atlanta, Georgia, United States of America; University of Surrey, School of Veterinary Medicine, UNITED KINGDOM

## Abstract

2020 saw the continuation of the second largest outbreak of Ebola virus disease (EVD) in history. Determining epidemiological links between cases is a key part of outbreak control. However, due to the large quantity of data and subsequent data entry errors, inconsistencies in potential epidemiological links are difficult to identify. We present chainchecker, an online and offline shiny application which visualises, curates and verifies transmission chain data. The application includes the calculation of exposure windows for individual cases of EVD based on user defined incubation periods and user specified symptom profiles. It has an upload function for viral hemorrhagic fever data and utility for additional entries. This data may then be visualised as a transmission tree with inconsistent links highlighted. Finally, there is utility for cluster analysis and the ability to highlight nosocomial transmission. chainchecker is a R shiny application which has an offline version for use with VHF (viral hemorrhagic fever) databases or linelists. The software is available at https://shiny.dide.imperial.ac.uk/chainchecker which is a web-based application that links to the desktop application available for download and the github repository, https://github.com/imperialebola2018/chainchecker.

## Introduction

2020 saw the continuation of the second largest outbreak of Ebola in history.

Contact tracing and determination of epidemiological links are key pillars of EVD outbreak control and are an important aspect of the multi-faceted response. However, due to the large quantity of data and different data sets, as well as subsequent data entry errors, inconsistencies in potential epidemiological links are difficult to identify. There are number of existing tools to visualise transmission links, particularly *epicontacts*, an R package to examine transmission links and Go.Data, a tool to collect and visualise contact data [1, 2]. However, none of the above currently verify transmission links from routinely collected data such as symptom onset date or date of death.

The chainchecker online and desktop applications allow the user to visualise and verify transmission links and chronological data. The focus is on user-defined values which are incorporated to a logic set to verify transmission links. The aim is that inconsistencies due to mis-entered data or conflicting information can be highlighted easily and quickly. The results can then be visualised interactively. These tools have been actively used in the field as part of ongoing outbreak investigations.

## Implementation

The chainchecker applications are implemented as R shiny applications either run on a web server or using R portable. They have dependencies on the shiny, shinythemes, plotly, ggplot2, epicontacts, tibble, dplyr, shinycssloaders, lubridate, data.table, magrittr, igraph, DT, network, GGally, sna, intergraph and htmlwidgets packages [1, 3–19]. The R software itself, as well as these required packages, can be obtained from the CRAN website at [http://cran.r-project.org]. The code is hosted on GitHub at [https://github.com/imperialebola2018/chainchecker].

The app establishes a framework for estimation and verification of epidemiological links between EVD cases. Verification depends partly on the calculator logic stated in Box 1. This helps highlight inconsistencies in dates that the user may then investigate. The calculator logic has preset values of e.g. time from symptom onset to death based on past EVD outbreaks based on Valasquez et al. [20]. However, these can be updated and adjusted by the user to best reflect the outbreak of interest.

**Box 1. Decision and calculation steps to provide a window of possible exposure to Ebola virus.**

The steps rely either on a date of death or reported date of symptom onset. Where possible, physical symptoms are also included to refine the exposure window calculation. Parameters such as minimum incubation period have preset values informed by previous outbreaks; however, these can be adjusted by the user.

1. *If date of death is available*. *Estimated onset = date of death—time from onset to death*. *Then go to 5*.
  - *If date of death is unavailable*, *go to 2*.
2. *If individual was bleeding at reported onset*. *Estimated onset = reported onset—bleeding correction factor*. *Then go to 5*.
  - *If they were not bleeding go to 3*.
3. *If individual had diarrhoea at reported onset*. *Estimated onset = reported onset—diarrhoea correction factor*. *Then go to 5*.
  - *If they did not have diarrhoea go to 4*.
4. *Estimated onset = reported onset*.
5. *Earliest exposure date = estimated onset—maximum incubation period*.
6. Latest exposure date = estimated onset—minimum incubation period

The applications include a number of features to help understand, visualize and verify possible epidemiological links. These include:

- Timeline: A timeline for individual case calculated using the calculator logic from available information such as date of death.
- Upload: An option to upload data in the format of the viral hemorrhagic fever (VHF) database. This tab also includes a template and minimal example which we shall explore in the following section.
- [offline version] A data entry page. This allows the user to correct or append to the existing uploaded data facilitating database management.
- Exposure windows: Timelines and estimated exposure windows for the uploaded data set. This allows the user to compare the exposure windows of possible linked cases.
- Transmission tree: A transmission tree for the proposed epidemiological links with inconsistent links highlighted. Inconsistencies are assessed through the estimated exposure windows of the infectee with respect to the infector.
- Cluster plots: A cluster analysis to highlight linked cases.
- [offline version] Visualisations of hospital stay by case or by health facility in order to examine the possibility of nosocomial transmission.

## Examples

### Template example

We illustrate a possible workflow for visualising and verifying transmission links using dummy data. These data are also available to download as a template. We then illustrate a more complex example taken from the “Ebola simulation part 2: outbreak reconstruction” from RECON learn, https://www.reconlearn.org/post/practical-ebola-reconstruction.html.

The default of this timeline is to display the symptom onset dates as reported. However, we may also select an option to estimate the symptom onset dates given the calculator logic in Box 1. Next, examine the transmission tree. The default visualization uses the symptom onset dates as reported although, as before, we may choose to use the estimated symptom onset dates. Fig 2 shows the dates as reported.

**Fig 1:**
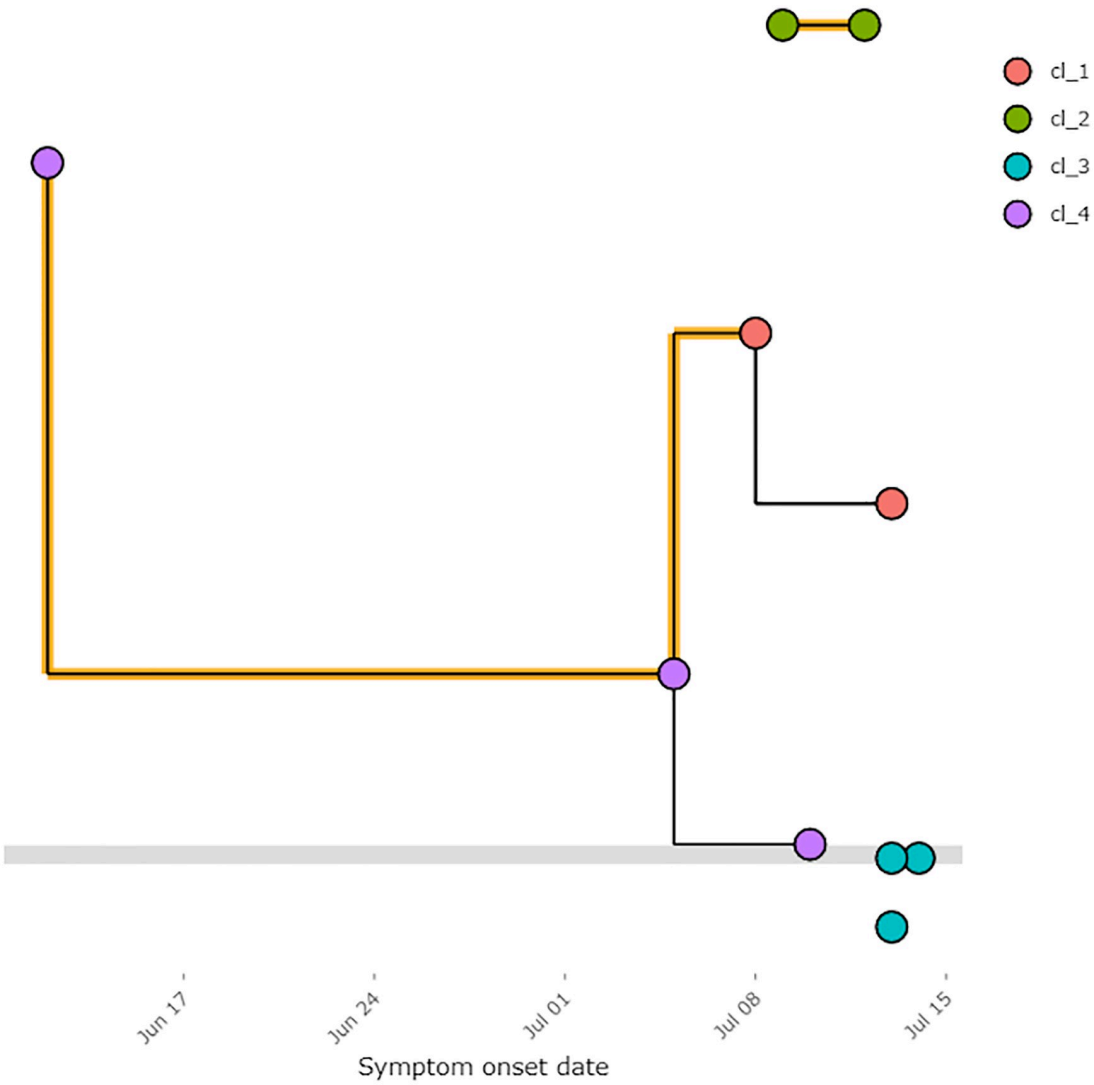
Basic transmission tree with symptom onset dates as entered. These are visualised using the online shiny app using data from the template. Note, the colour of the node can depend on any entered field from the linelist, as specified by the user, in this example it depends on the cluster number. This example is available to download in templates from the online shiny application.

In Fig 1 we see some transmission links have been flagged in orange as inconsistent. To understand why, we may download the inconsistencies as a.csv file which will state which links are inconsistent, the reason why and the symptom onset dates for the infectee and the infector. We may also go back to the exposure windows and specify the unique ID of two individuals to visualise why they may have been flagged. In this example, EG0 and EG1 we see the transmission link between the two has been flagged because EG0 died before EG1 was exposed. This could indicate either funeral transmission or an inconsistency in the data.

In Fig 1, we also note that three cases are unconnected. These are stacked by symptom onset date below the grey line, indicating that they do not appear in the list of contacts and that further investigation may be needed in order to epidemiologically link them to existing cases. Finally, we may adapt the tree visualisation to show other variables of interest. These could include gender or location from the linelist or transmission type from the list of contacts.

Finally, we highlight the cases by cluster. In this example, we find four clusters where all cases without epidemiological links are defined to be in one cluster. We allocate clusters by the fast greedy modularity optimization algorithm implemented by Clauset, Newman, and Moore with node size relative to the number of connections a case has to other cases [21]. The cluster information tab allows the user to filter meta-data by cluster id, significantly reducing time spent searching for and pooling data from related cases.

### Further example

**Fig 2:**
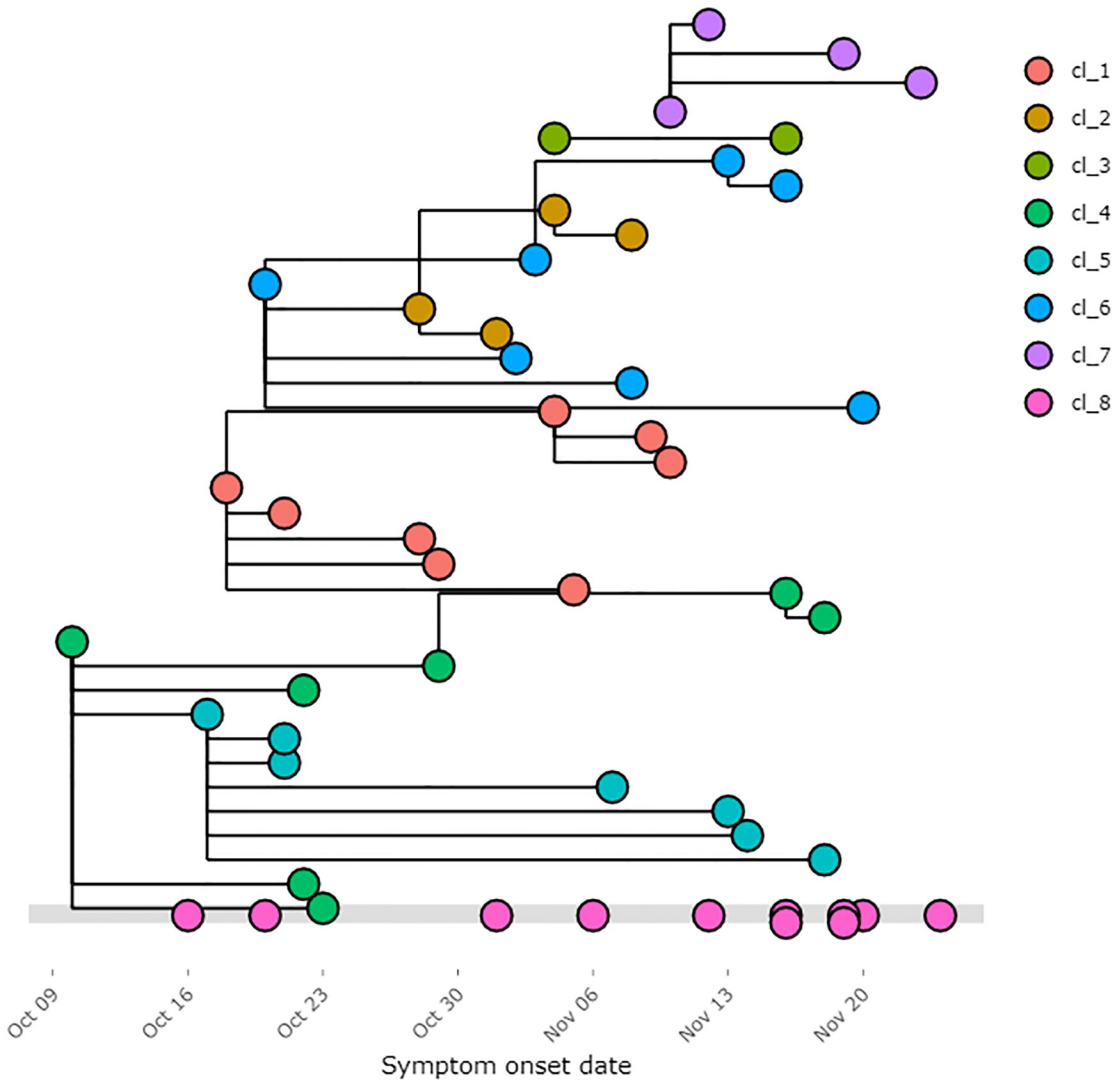
Extended transmission tree with symptom onset dates as entered. These are visualised using the online shiny app using data from RECON learn. Note, the colour of the node can depend on any entered field from the linelist, as specified by the user, in this example it depends on the cluster number.

We further illustrate the application with the example shown in Fig 2.

The example in Fig 2 illustrates the utility of the application in being able to quickly and efficiently visualise complex datasets. The unlinked cases are shown on the grey line and may appear to be increasing in this example, a common feature of outbreaks as more recent cases have yet to be investigated fully. Individual clusters are highlighted and can be explored further in the cluster tabs of the online application. This can help identify common features of transmission and significantly expedite the qualitative investigation of related case narratives. Finally, the proposed links themselves can be examined to check they follow in terms of exposure and contact dates.

## Discussion

Chainchecker enables the user to perform visualisation, data curation and verification of linelist data for EVD. It has been successful in producing interactive transmission chain visualizations whilst highlighting possible discrepancies. Similarly, the latest features related to cases clustering and visualising nosocomial transmission are highly relevant for questions surrounding healthcare related transmission events.

The cluster output is also of particular use in rapidly highlighting groups of related cases which may require further qualitative investigation. This can assist the user in establishing the narrative of a single cluster and identify common clustering patterns, for example whether they are generally formed of family members, attendees at specific events or locations.

The aim of chainchecker is to facilitate the user proposing and verifying epidemiological links. To this end, parameter values used in the calculator logic can be adjusted by the user, as can all of the visualisations. Similarly, data entries can be edited and managed by the user. This means that the chainchecker applications have no predictive potential. Indeed, it is only recently that freely available software has been developed to reconstruct and predict transmission links using a variety of data types [22]. However, prediction requires more data, a larger variety of data types, usually including genomic data, and can be time-consuming. Whilst chainchecker cannot predict, it provides a fast and user-driven tool to improve contact data collection and visualisation in the field.

The applications were built to be user friendly tools for quick analysis. However, the framework of utilising shiny applications to turn coding bases into more general tools is extremely flexible, particularly for outbreak analytics. With a more complex data requirement, chainchecker could be integrated into other workflows in order to estimate reproduction numbers and predict future case numbers. Alternatively, the approaches within chainchecker could be adapted for other, existing, software such as go.data. With a wealth of outbreak analytical packages within R, there are opportunities to consolidate approaches both in R and through, potential more accessible, shiny applications.
